# Part-Based and Configural Processing of Owner's Face in Dogs

**DOI:** 10.1371/journal.pone.0108176

**Published:** 2014-09-24

**Authors:** Elisa Pitteri, Paolo Mongillo, Paolo Carnier, Lieta Marinelli, Ludwig Huber

**Affiliations:** 1 Department of Comparative Biomedicine and Food Science, University of Padova, Legnaro, PD, Italy; 2 Messerli Research Institute, University of Veterinary Medicine Vienna, Medical University of Vienna, and University of Vienna, Vienna, Austria; Université Pierre et Marie Curie, France

## Abstract

Dogs exhibit characteristic looking patterns when looking at human faces but little is known about what the underlying cognitive mechanisms are and how much these are influenced by individual experience. In Experiment 1, seven dogs were trained in a simultaneous discrimination procedure to assess whether they could discriminate a) the owner's face parts (eyes, nose or mouth) presented in isolation and b) whole faces where the same parts were covered. Dogs discriminated all the three parts of the owner's face presented in isolation, but needed fewer sessions to reach the learning criterion for the eyes than for both nose and mouth. Moreover, covering the eyes region significantly disrupted face discriminability compared to the whole face condition while such difference was not found when the nose or mouth was hidden. In Experiment 2, dogs were presented with manipulated images of the owner's face (inverted, blurred, scrambled, grey-scale) to test the relative contribution of part-based and configural processing in the discrimination of human faces. Furthermore, by comparing the dogs enrolled in the previous experiment and seven ‘naïve’ dogs we examined if the relative contribution of part-based and configural processing was affected by dogs' experience with the face stimuli. Naïve dogs discriminated the owner only when configural information was provided, whereas expert dogs could discriminate the owner also when part-based processing was necessary. The present study provides the first evidence that dogs can discriminate isolated internal features of a human face and corroborate previous reports of salience of the eyes region for human face processing. Although the reliance on part-perception may be increased by specific experience, our findings suggest that human face discrimination by dogs relies mainly on configural rather than on part-based elaboration.

## Introduction

Dogs are largely exposed to human faces and their aptitude to look at them is evident in many different situations [1]–[5]. Dogs are also very skilful in processing human faces presented as two-dimensional stimuli: they discriminate human faces from those of other species [6], their owner's from another known person's face [7] and even different face expressions [8]. Moreover, dogs exhibit characteristic looking patterns when viewing human face pictures, including both an eye bias [9] and a left gaze bias [10]. Some insight about how exactly the processing of human faces is carried out comes from eye tracking studies. For example, there seems to be no difference in looking time between novel and familiar pictures of human faces when the stimuli are presented upside-down, indicating the presence of an inversion effect, which deteriorates discriminative responses [11]. Other recent studies found that dogs inspect 2D face images by focusing on their informative regions [12] and that facial inversion and familiarity with the person affect the scanning behaviour of dogs [9]; in particular the eye region of upright faces gathers longer total duration and greater relative fixation duration than that of inverted stimuli and faces belonging to known persons are more fixated than those belonging to strangers. These findings suggest that dogs are likely to recognize human faces in photographs and this hypothesis is also supported by Adachi et al. [13] who demonstrated that dogs formulate expectations regarding the visual aspect of the owner's face looking longer when the 2D image of the face presented contradicted the auditory stimulus (an unfamiliar voice).

Since dog's scanning behaviour is affected by facial inversion [9], [11] and in humans inverted facial image has to be processed mainly element by element [14], [15] another crucial unsolved issue is whether dogs can process a face by its elements, and even most important, whether they can perceive and discriminate face elements at all. To date no data are available, except from a study by Huber et al. [7] where it was found that the discrimination of human faces is harder when only the inner parts of the face (eyes, nose and mouth) are visible. To this respect it would be also important to investigate whether some features could be more relevant for face discrimination in dogs, since in human literature it has been established that there is a hierarchy of features in terms of their diagnostic value to face processing [16]. In particular the eyes are the most important feature involved in individual identification [17], receive more visual attention than other areas of the face [18] and are perceptually more discriminable than nose or mouth [19], [20]. Eyes could be the most relevant feature also for dogs, since it is the region they mostly look at when viewing human faces [9].

Our knowledge about mechanisms underlying face processing comes primarily from human studies, most of which support the holistic nature of the process, whereby faces are perceived as whole, rather than as the sum of their component parts. One aspect that has traditionally been tackled by these studies is the extent to which configural information (i.e. the spatial relationship between parts) as opposed to information about the parts themselves contributes to the processing of faces. Indeed, while face discrimination has proven to be configuration-dependent [15], to the point that *configural* and *holistic* are sometimes used interchangeably, e.g. [21], [22], others have stressed that discrimination also necessitates information about face parts [23]. Previous approaches have examined the importance of part and configural information by selectively removing the possibility to rely on such information, for instance by examining the ability to discriminate faces basing on parts only [24], or through systematic manipulation of face image to reduce or eliminate some aspects of the information [25]. More or less sophisticated manipulations have been used in different species and in the present study, as a first approach, we chose two that reduce as specifically as possible the elaboration of configural or elemental information, respectively inversion and blurring, e.g. [26], and one that allows to alter both of them, that is scrambling. Inversion is the upside-down presentation of stimuli and, while not removing information, it impairs configural processing [27], making a face harder to discriminate by both humans [14], [28] and other primates, e.g. [29]–[32]. The inversion effect on face visual inspection has been already tested in dogs [9], [11] but an active (approach and touch) discrimination tasks has never been used to this aim. However, active choice would give a more incontrovertible evidence of dogs' ability to discriminate faces and add some possible explanations of the differences in gazing patterns observed. As opposed to inversion, blurring affects part-based more than configural processing, at least at intermediate levels of blurring [33]. Notably, this does not seem to affect face processing abilities in humans and macaques [34], while face processing by chimpanzees is impaired when individual features are blurred through pixilation [25]. Scrambling affects mainly configural processing, but even part perception is affected to an extent that depends on the magnitude of the manipulation itself, e.g. [15], [35]. Visual discrimination is impaired by scrambling in humans [15] and rhesus monkeys [18] when looking at human faces; in contrast, pigeons are still able to discriminate cartoon characters [36], [37] and photographs of people [38] even if scrambled in tiny fragments.

Another controversial issue regards the extent to which face processing abilities are due to experience. Accumulating evidence suggests for neonates an ability to specifically process faces [39]–[42] and a face-specific heritability for holistic processing has been demonstrated in twins [43]. Nevertheless, the acquisition of fine face-processing abilities requires years of exposure to faces (as reviewed in de Haan et al. [44]) and experience is required in order to apply established mechanisms to different face subsets [45]–[47]. The importance of experience is especially evident in the processing of hetero-specific or other-race faces. Face processing by humans improves after specific training or after exposure to other-race individuals [48]–[50] and a disadvantage in the discrimination of human faces by chimpanzees was cancelled out or indeed overturned by intensive exposure to humans [51]. Differences in visual processes underlying conspecific or heterospecific face discrimination are reported in both humans and monkeys who show different patterns of eye movement depending on the species' affiliation of the faces [34]. Species-specificity in inspecting conspecific versus human face images was reported also in dogs [9]–[11]. Prior experience about certain face category could influence the distribution of dogs' gaze fixations directed at the specific face region, reflecting a different viewing strategy/sensitivity to sample relevant facial information from different species [52]. Therefore, it may be that the level of experience with heterospecific faces influences the relative recruitment of configural and part-based mechanisms. Besides, specific training could result in the use of strategies not normally used in natural conditions e.g., focusing on a single local region of the human faces, as argued for chimpanzees by Pascalis and Bachevalier [53].

In the present study we conducted two experiments to assess the mechanisms underlying human face processing by dogs. In Experiment 1 we examined whether dogs can discriminate human face parts presented in isolation and if so whether one of them could be more relevant for face discrimination. Experiment 2 investigated the relative contribution of part-based and configural processing in discrimination of human faces and the influence of specific experience on the reliance on such mechanisms.

## Experiment 1

Human and nonhuman primates studies suggest that the eyes are the most important facial feature involved in face processing. Even if dogs demonstrated to discriminate pictures representing different expressions of their owner and their owner's from another person's face, their ability to perceive face parts is supposed, but not yet clearly proven. Therefore the preliminary aim of this experiment was to investigate the ability of dogs to perceive and discriminate internal face parts (eyes, nose, mouth) belonging to the owner, presented in isolation. Moreover, the role of face parts for face discrimination was assessed by training dogs to discriminate their owner's whole face with one of the three parts covered (eyes, mouth or nose covered) and finally to discriminate the normal whole face as a control condition. By comparing the performances of these trainings we assessed whether any of the parts was more important than the others for the discrimination of the human face.

### Methods

#### Ethics statement

Owners participated in the experiments of the present study on a voluntary basis; they signed a consent form and agreed to have their portraits published in this paper. This study was performed in compliance with relevant laws in Austria and gained the approval of the Ethics Committee of the University of Veterinary Medicine, Vienna (No. 10/04/97/2013).

#### Subjects

Dogs and their owners were recruited from dog owners living in or around Vienna to participate in this study. All dogs were pets, living with the owner and had daily contact with humans. Dogs had basic obedience training and they had been working with the touch screen (although with a slightly different apparatus), but had neither seen the visual stimuli used in this experiment, nor had been trained with other pictures that included human faces or parts of them. Dogs were on normal diet and they were unleashed during the entire procedure. Of the 12 dogs enrolled, seven completed all phases and were included in the study (N = 7; 4 females, 3 males; mean age ± SD  = 3.4±2.1). Due to a strong side bias the training of the remaining five dogs was terminated after 800 trials.

#### Experimental setting

**Figure 1:**
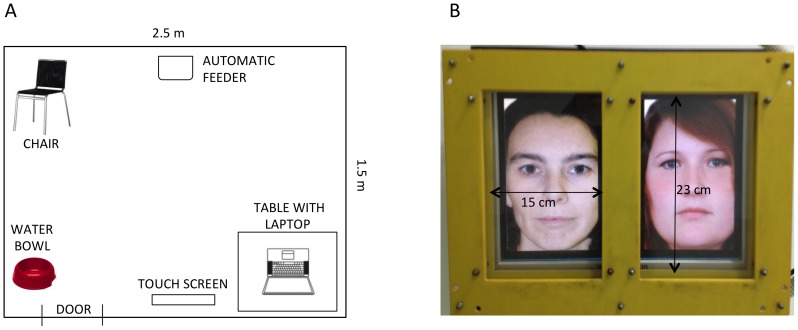
Experimental setting and detail of the touch screen. Details of the experimental setting showing (A) a schematic representation of the experimental room, illustrating the position of the touch screen, the laptop, the automatic feeder, the water bowl and the owner's chair and (B) a photograph of the touch screen, with dimensions of the hemi-screens; the pictures shown in the screen exemplify the presentation of stimuli, in this case the whole, uncovered faces of the owner and the stranger.

The study was conducted in an experimental room of the Clever Dog Lab at the Messerli Research Institute of the University of Veterinary Medicine of Vienna (size: 2.5 m×1.5 m; Fig. 1A). The testing apparatus consisted of a touch screen (40×31 cm; Fig. 1B) fixed to the wall in a flexible way so that its height could be adjusted to the height of the dog. It was built up of a commercial flat screen (resolution 1024×748 pixel) fixed on a metal plate. Dogs' responses were recorded by special pressure sensors that were connected with two separate acrylic glass panes (each 15×23 cm), which were fixed in front of the screen. The pressure sensors were linked to a remote control, which was connected via an interface to the computer. A laptop was used to control the presentation of stimuli, the provision of food and the registration of responses. Reinforcement was administered in the form of small commercial dog food pellets, delivered by an automated feeding device (MannersMinder, Premier Pet, LLC, 14201 Sommerville Ct., Midlothian, VA 23113, USA) that was placed on the floor on the opposite side of the touch screen. Owners were always present in the experimental room during the experiment; they sat on a chair from where they could not see the stimuli, thus preventing them from unintentionally influencing the dogs' choice. The experimenter was also present in the room to control the correct progression of the procedure but she was unaware of which stimuli were being presented to the dog.

#### Stimuli

**Figure 2:**
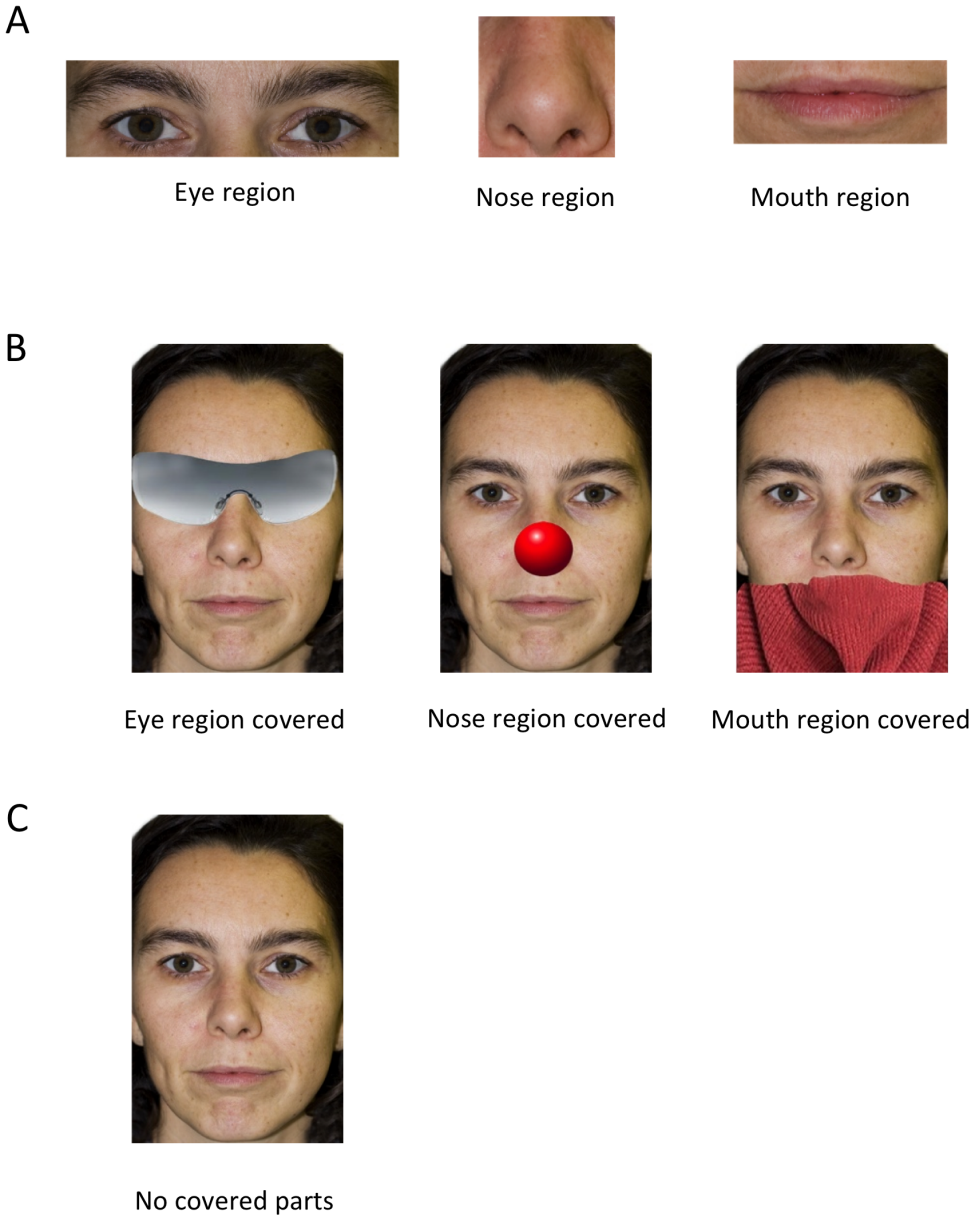
Example of the stimuli used in Experiment 1. Example of stimuli used in the Experiment 1, representing (A) face parts presented in isolation and (B) the whole face with single parts covered and with no parts covered (C).

The stimuli were made from a picture of the owner's face and a picture of a stranger person's face of the same gender as the owner. Since all participating owners were women, all pictures consisted of female faces. As the study tried to answer basic questions, only one picture of the owner and of a stranger were used throughout the experiment. This allowed a greater standardization of the stimuli, avoiding the introduction of variability that could have influenced discrimination processes (e.g. different expressions, poses, colours, shading, etc.). Moreover, testing for individual recognition was not the aim of the present experiment, therefore generalization to a set of pictures was unnecessary. To standardize their appearance as much as possible, pictures were taken in a photographic setting with a professional camera (Canon EOS 6D, Canon, Japan) and people were previously asked to wear no makeup or jewellery and to look straight into the camera with a neutral facial expression. The pictures were then processed using Adobe Photoshop CS4 Extended (11.0 Adobe Systems Inc. 1990–2008) to adjust lightning, contrast, size and to add a homogenous white background. The same software was used to create the stimuli of Experiment 1 and 2. The size of the pictures was adjusted to match that of the real heads as much as the width of the screen allowed (14.8 cm). Seven types of stimuli were created from this picture: three were a rectangular crop of the eyes, the nose or the mouth region, which appeared as a rectangular area on a white background; these regions had the same area across all people and maintained the same size as in the whole face (eyes  = 32,3 cm^2^; nose or mouth  = 9,61 cm^2^, Fig. 2A). Other three stimuli represented the whole face, where images of sunglasses, of a clown-nose or of a scarf were superimposed to cover either the eye region, the nose region or the mouth region, respectively (Fig. 2B). The last stimulus was the picture of whole face with no parts covered (Fig. 2C).

#### Procedure

The presentation of the stimuli, their position (randomly left or right) and the data collection (session duration, choice) were done using the software DogIT (written by Dietmar Schinnerl, Graz, Austria). All sessions consisted of 20 trials, in which one positive (the owner's face or face part, S+) and one negative stimulus (the stranger's face or face part, S-) were simultaneously presented to the dog at a distance of approximately 100 cm. The touching (with the nose) of S+ was followed immediately by a short high-pitched tone, disappearance of both stimuli from the screen and provision of food reward. The touching of S- was immediately followed by a short low-pitched tone and the screen turned into red for 3 s. Wrong choices were followed by correction trials (the presentation of the same stimuli and in the same position as in the previous trial, until the correct response was delivered). Correction trials were not taken into account when summing up the number of trials per session or the number of correct choices within a session. The learning criterion to successfully complete a phase and proceed to the following one was set at 85% correct choices in three consecutive sessions (i.e. 51 correct trials out of the last 60).

The owners came with their dogs to the sessions twice a week. On a single day, dogs completed 6 sessions in 30 to 40 min, with a 5-min break after the third session.

#### Pre-training

All dogs were already trained to choose the stimulus by touching the monitor with their nose, but they were used to a slightly different apparatus, the one described in Range et al. [54]. Therefore, dogs were accustomed to the new apparatus and the new feeder, by training them with simple shapes (circle or square) on a black background, in a two-way conditioned discrimination procedure until they reached the learning criterion (see above). This required an average of 4.7±2.1 (mean ± SD, range 3–8) sessions.

#### Training phases

The dogs underwent six training phases, one for each stimulus type (i.e. eye, nose or mouth regions only and eye, nose or mouth regions covered). Once dogs reached the learning criterion with a given stimulus type, they proceeded to the next training phase with a different stimulus type. Three dogs started with the isolated parts and then proceeded to the faces with covered parts; the other four dogs started with the faces with covered parts and then proceeded to the isolated parts. The overall sequence was different for each dog (Table 1). As a control condition, all dogs underwent a last additional training aimed at discriminating the whole face with no covered parts. The experiment was terminated as soon as the dog completed the control condition.

**Table 1:** Sequence of isolated and covered face regions presented to each dog in the training phases of Experiment 1.

	Order of presentation					
Dog ID	1st	2nd	3rd	4th	5th	6th
**1**	E-R	N-R	M-R	E-R covered	M-R covered	N-R covered
**2**	M-R	E-R	N-R	N-R covered	E-R covered	M-R covered
**3**	N-R	M-R	E-R	M-R covered	N-R covered	E-R covered
**4**	E-R covered	N-R covered	M-R covered	M-R	N-R	E-R
**5**	M-R covered	E-R covered	N-R covered	N-R	E-R	M-R
**6**	N-R covered	M-R covered	E-R covered	M-R	N-R	E-R
**7**	E-R covered	M-R covered	N-R covered	E-R	M-R	N-R

Once dogs reached the learning criterion with a given stimulus type, they proceeded to the next stimulus until the sixth training phase.

E-R  =  eyes region, N-R  =  nose region, M-R  =  mouth region.

#### Data collection and statistical analysis

Data regarding the duration of sessions and the choice (S+ or S−) made by dogs at each trial were recorded. The total number of sessions to reach the learning criterion in each phase was considered as variable to investigate the difficulty encountered by dogs to acquire the tasks.

A linear mixed model was used to assess whether any of the owner's isolated face parts was more easily discriminated than the others. The total number of sessions required to reach the learning criterion was used as a dependent variable. The type of stimulus (eyes, nose, mouth) was used as a within-subjects factor, and, to control for an effect of the order of presentation of the different stimulus types, this was also included in the model, as was the interaction order*stimulus type. To account for the repeated measures, the dog's identity was fitted in the model as a random factor. The procedure was followed by pairwise contrasts, with Bonferroni correction for multiple comparisons. A linear mixed model was also used to assess the relative relevance of face parts for the discrimination of the owners' face. Again, the number of sessions to criterion was used as the dependent variable, on which a natural logarithm transformation was applied to obtain a normal distribution. The explanatory variable was the type of stimulus (covered eye region, covered nose region, covered mouth region, no part covered). Bonferroni-corrected pairwise contrasts were performed between performances with one part covered vs. performance with none of the parts covered, which was taken as a control condition. Since the face with no covered parts was invariably the last stimulus that was presented, the order of presentation could not be fitted in this model; however a separate model was built, where data from the face with no covered parts was excluded, to assess the effect of the order of presentation of faces with a covered part. The dog's identity was fitted in the models as a random factor, to account for the repeated measures.

All the analyses were performed with Statistical Analysis System software (SAS Institute Inc SAS/STAT 9.2 User's Guide. Cary, NC: SAS Institute Inc; 2008) and the statistical significance was set at 5%. Data are presented as mean ± SD unless otherwise stated.

### Results

The duration of training sessions ranged from 2.2 to 13.1 min with an average of 4.7±1.6 min.

Dogs showed a certain inter-individual variability in the number of sessions needed to acquire the task with both isolated parts (min-max: eye region  = 12–19, mouth region  = 12–36, nose region  = 21–34) or whole faces (min-max: eye region covered  = 4–21, mouth region covered  = 3–14, nose region covered  = 4–24, no part covered  = 3–7).

**Figure 3:**
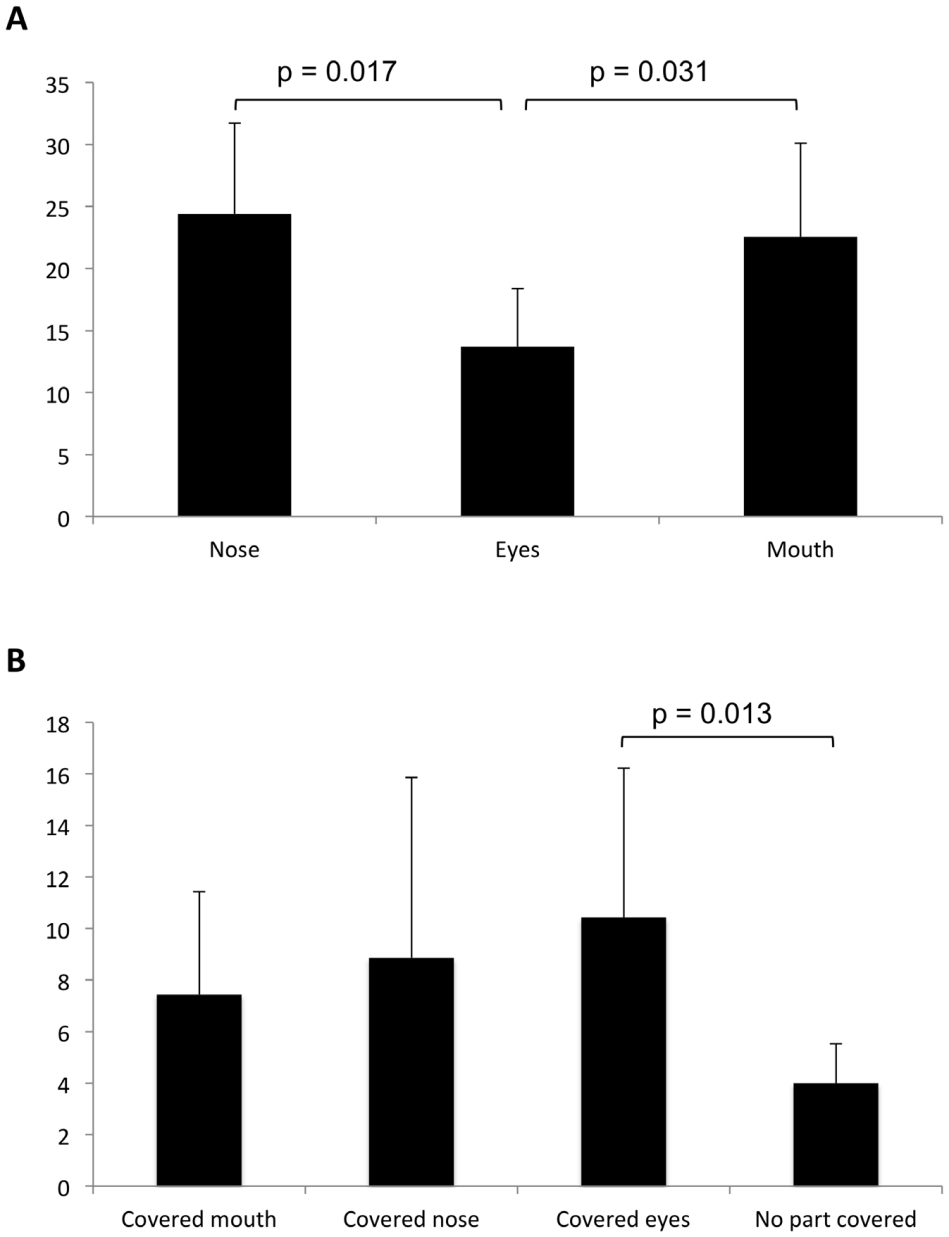
Sessions needed to reach the learning criterion in the training phases of Experiment 1. Mean ± SD number of sessions needed by dogs to reach the learning criterion when discriminating between (A) the owner's and stranger's face isolated parts and (B) faces with covered parts. Bonferroni corrected pairwise comparisons after generalized linear mixed model.

When dogs were presented with isolated face parts, their performance was significantly affected by the type of stimulus (F_2,5_ = 11.97, p = 0.012) and not by the order of presentation (F_5,5_ = 0.80, p = 0.59) or by the order*stimulus type interaction (F_8,5_ = 4.25, p = 0.064). Specifically, discrimination of the eye region required fewer sessions compared to both the mouth and the nose region, while no significant difference was found between the nose and mouth regions (Fig. 3A).

The type of stimulus also had an effect on the dogs' performance when whole faces were presented (F_3,24_ = 3.59, p = 0.028), since discrimination was significantly faster for the face with no covered parts than when the eyes region was covered, but not than when the nose region or the mouth region was covered (Fig. 3B). No effect was found for the order of presentation of faces with covered parts (F_5,3_ = 0.96, p = 0.549), or for the order*type of stimulus interaction (F_10,5_ = 0.198, p = 0.978).

### Discussion

This first experiment revealed an ability of dogs to discriminate all the three internal parts of the owner's face presented in isolation, with an advantage for the eye region over both the nose and the mouth regions. Discrimination of the eye region could have been easier merely because it covers a bigger area and is richer in features and details, such as colours and shapes. Alternatively, or in addition, eyes may have been easier to discriminate because they are the most salient feature of the face.

The dogs' performance with faces in which the parts were covered provides indication for choosing between these two hypotheses, which are not mutually exclusive since quantity of details could be embedded also in salience. The dogs were readily able to discriminate the owner's whole faces in the control condition and no difference in discriminability was found between this condition and those where the nose or mouth regions was hidden. It would not be correct to conclude that these regions do not convey useful information but simply we could not reliably measure their effect with our experimental design. Conversely, masking the eyes region significantly disrupted face discriminability compared to the whole face condition. If the mere quantity of unspecific perceptual details and information was the only reason of difference in discriminability we would expect also the absence of nose or mouth regions information to have an effect on the ease of face discrimination. Moreover, the face with eyes covered is still very rich in perceptual details and carries substantial information, including some external cues (face profile, hair), which dogs can effectively use to discriminate between human faces [7]. However, if this was the case no difference in discriminability would have been found whatever internal face region was masked. Therefore the eye region seems to have a special role in human face processing of dogs, corroborating the findings of Somppi et al. [9] and supporting the hypothesis for a role of the eyes region in a global mechanism of face processing [34], [55], [56].

## Experiment 2

In the Experiment 1 dogs discriminated both the face parts presented in isolation and the whole faces with one part covered, showing that they can use the information of the parts to discriminate the human faces presented in the pictures. This second experiment examined the relative contribution of part-based and configural processing in the discrimination of the owner's face from a stranger's face by manipulating the type of information available. Furthermore, we assessed the influence of specific experience on the reliance on such mechanisms by comparing dogs enrolled in the first experiment and a new group of dogs, naïve to the procedure and to the pictures.

### Methods

#### Subjects

The same seven dogs that completed Experiment 1 participated in Experiment 2. In addition, to verify whether there could be an influence of the training that dogs underwent during Experiment 1 on performance in Experiment 2, other seven dogs were recruited (naïve group, 6 females, 1 male; age = 4.9±1.9) and compared with the seven dogs that completed Experiment 1 (expert group). Naïve dogs had analogous obedience training and the same experience with the touch screen procedure as the expert group. The overall sample was composed of 14 adult family dogs of 4.1±2.1 years of age.

#### Experimental setting and general procedure

The experimental setting and the general procedure were the same as in Experiment 1.

#### Stimuli

**Figure 4:**
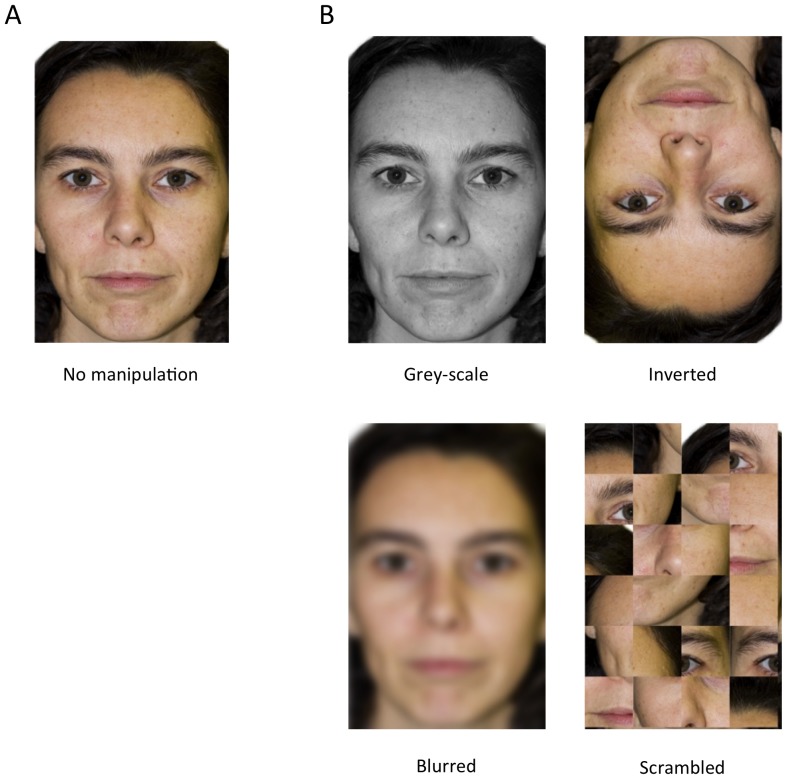
Example of the stimuli used in Experiment 2. Example of stimuli used in Experiment 2, representing (A) the whole face in frontal view without manipulations and (B) the whole face with manipulations used in the test trials.

The stimuli used in the training phase of naïve dogs were a photograph of the owner's face in frontal view, realized in the same conditions as for the Experiment 1 and the same photograph of the stranger used in the first experiment (Fig. 4A). Test stimuli (Fig. 4B) were a grey-scale, a blurred (filter blurring r = 8.0 pixel), an inverted (180° rotation) and a scrambled (randomly rearranged squares of 2.7 × 2.9 cm; 102,1 × 109,6 pixel) version of the same photographs used in training for both groups. The grey-scale manipulation was added to assess the importance of specific experience in the discrimination, since expert dogs did not receive training to this respect.

#### Training

Prior to starting Experiment 2, the seven dogs belonging to the naïve group underwent the pre-training phase and the control condition training, in which whole faces with no manipulation were presented, as described in Experiment 1. Once the learning criterion of 85% correct choices in 3 consecutive sessions was reached, naïve dogs proceeded to the test phase. In contrast, dogs of the expert group started with the test phase as soon as they had completed Experiment 1.

#### Test

Test sessions were made up of 16 training trials and 4 test trials. In training trials, non manipulated photographs of the owner's face and of the stranger's face were presented. Only if the dogs performed sufficiently well in the training trials (85% correct or more), the sessions were included in the analysis of test performance. All tested dogs fulfilled this requirement. In test trials, the four test stimuli belonging to the owner (S+) and the stranger (S−) were presented in random order, once every five trials, beginning with trial number five. Dogs were always rewarded in the test trials regardless of their choice and no correction trials were administered. Twelve test sessions were performed in two different days, for a total of 48 test trials for each dog, i.e. 12 test trials for each of the four test stimulus types.

#### Data collection and statistical analysis

Data regarding the duration of sessions, the choice (S+ or S−) made by dogs at each trial, and the side (left, right) of the chosen stimulus were recorded. A paired sample *t*-test was run to analyse the differences between expert and naïve dogs in the mean number of sessions required to acquire the learning criterion in the whole face training phase. A logistic regression model [57] was then used to analyse whether the probability of choosing the owners' face picture in the test phase was significantly affected by the image manipulation and by the dog's experience in the procedure. The dependent variable was a dichotomous categorical variable representing the dog's choice at each presentation (1 =  owner's face; 0 =  stranger's face), whereas the explanatory variables were the experience group (expert or naïve) and the type of manipulation (grey-scale, blurred, inverted, scrambled); moreover, the side of presentation of owner's face (left or right) was added to the model as a fixed effect and the dog (N = 14) was added as a random effect.

To asses whether a prevalence for the owner's face or the stranger's face was present in our sample, we performed a right-tailed Student's *t*-test on the dogs means for the choice (1 =  owner's face; 0 =  stranger's face) expressed in the 12 test trials of each of the four manipulations, testing the null hypothesis H_0_ that the mean was equal to or lower than 0.5. Also we computed the probability that the true mean for the choice was in the range between 0.501 and +∞. On the basis of the results obtained by the logistic regression model, the right-tailed Student's *t*-test was performed on the overall sample in the test manipulations in which no experience effect was found, otherwise was performed separately for the two groups (expert and naïve).

### Results

Sessions of the training phase of naïve dogs had an average duration of 5.1±2.3 min. As expected, naïve dogs reached the learning criterion in the non-manipulated face training phase in significantly more sessions than expert dogs had done in the corresponding phase of Experiment 1 (4.0±1.5 vs. 18.7±7.5; t_12_ = −5.058, p<0.001).

**Figure 5:**
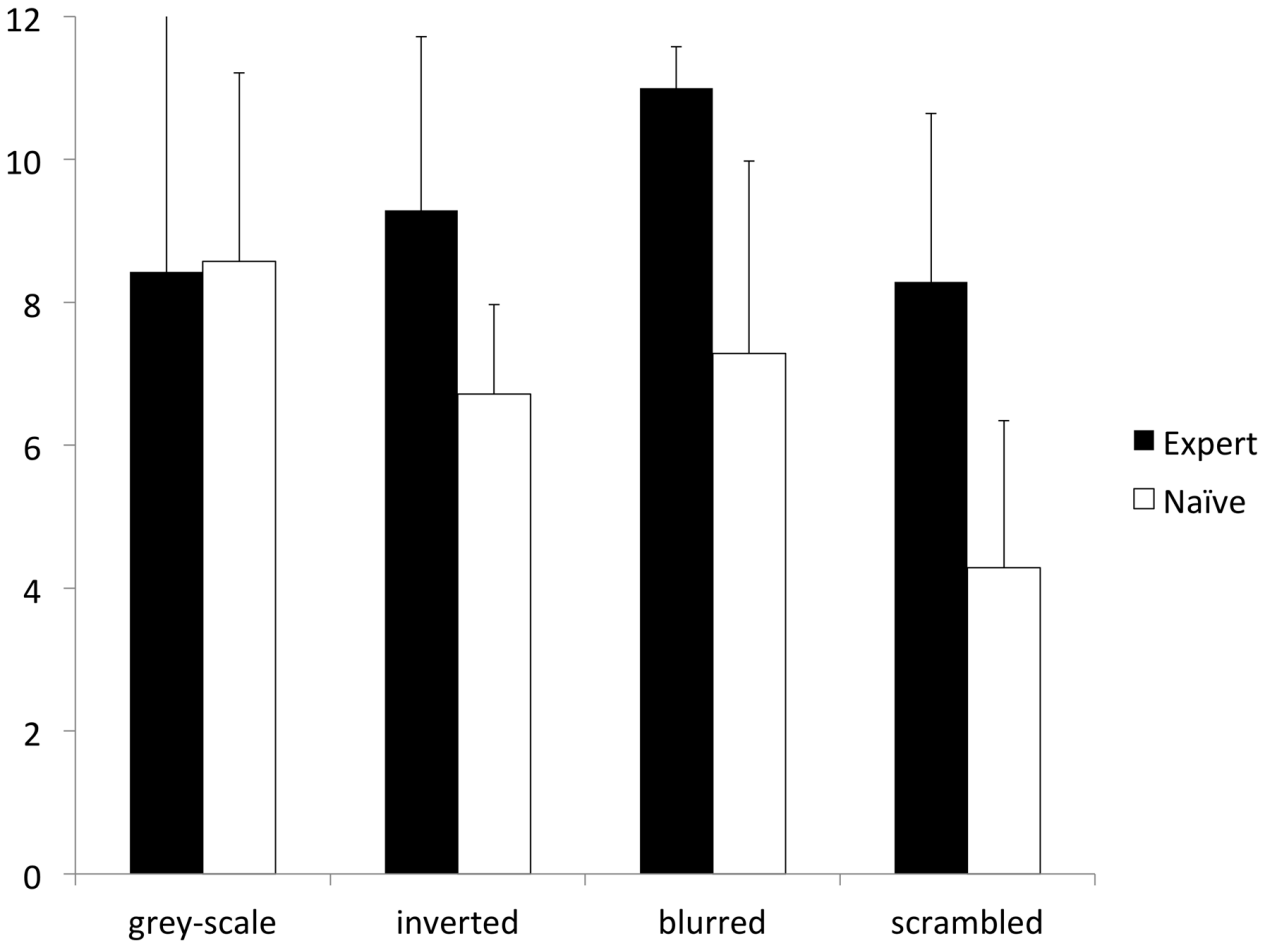
Choices in the test trials of Experiment 2 by expert and naïve dogs. Mean ± SD number of owner's face choices done by expert and naïve dogs with each type of manipulation in test trials of Experiment 2. Error bars represent the standard deviation from mean.

Sessions of the test phase had duration of 6.2±1.7 min. The binary logistic regression model indicated that the side of presentation of S+ and S− did not affect the dogs' choices in the test phase. In contrast, a significant effect was found for the type of manipulation, the experience group and the interaction between manipulation and group (Table 2). Comparing the two groups within each type of manipulation (Fig. 5), expert dogs showed a higher probability than naïve dogs of choosing the S+ in the blurred (F_1,651_ = 10.83, p = 0.001) and in the scrambled condition (F_1,651_ = 12.81, p<0.001), while no statistically significant difference was found in the inverted condition (F_1,651_ = 3.10, p = 0.079). The two groups performed almost identically in the grey-scale condition (F_1,651_ = 0.02, p = 0.899).

**Table 2:** Binary logistic regression model showing the effect of side of presentation, experience group, type of manipulation and interaction between group and type of manipulation on choices of the owner's face (S+) in the test trials of Experiment 2.

Effect	OR	95% CI	p
Experience group	2.555	1.452–4.495	0.0012
			
Type of manipulation	2.214	1.386–3.535	<0.0001
Experience Group * Type of manipulation	-	-	0.0041
Side of presentation of S+	1.102	0.783–1.550	0.5787
			

OR  =  odds ratio, CI  =  confidence interval.

In the grey-scale condition, a right-tailed *t*-test could not reject the null hypothesis that the mean of choices expressed by dogs as a group in the 12 test trials was less than or equal to 0.5 (t_13_ = 3.08, p = 0.004) indicating that all dogs significantly chose the owner above chance level. In the blurred condition both expert and naïve dogs chose the owner significantly above chance level (expert: t_6_ = 22.91, p<0.001; naïve: t_6_ = 2.90; p = 0.014), whereas only the expert group chose the owner above chance level in both the inverted (expert: t_6_ = 3.06, p = 0.011; naïve: t_6_ = 1.51; p = 0.091) and the scrambled condition (expert: t_6_ = 2.56, p = 0.021; naïve: t_6_ = −2.20; p = 0.965).

### Discussion

The aim of this experiment was twofold: to investigate in pet dogs a) the relative importance of configural and part-based processing and b) the influence of specific experience on the reliance on such mechanisms in the discrimination of human faces. Naïve dogs were able to discriminate the picture of their owner's face from that of a stranger only in the grey-scale and in the blurred condition, both of which provided configural information. Conversely, their performance dropped to chance level in the case of the inverted and the scrambled condition. Scrambling necessarily disrupts configural information and only the facial part information is available, even if not fully preserved (eyes, mouth and nose were all cut in the random rearrangement of the tiles). However, also inversion has been shown to facilitate processing of face parts [15], at the expense of the more efficient configural processing of upright faces. The performance of our naïve dogs therefore indicates that face discrimination could rely primarily on configural processing, at least without specific experience.

Expert dogs had been trained in Experiment 1 to discriminate their owners' isolated face parts and, accordingly, their performance in Experiment 2 was better than that of naïve dogs in those conditions that could be solved by part-based processing, namely scrambling and inversion. In this last condition, experience affected the dogs' ability to choose the owner only with a non-significant trend. However, this was sufficient to differentiate between groups, as expert, but not naïve dogs, were able to select the owner's face above chance level. Training experience had also an effect on the ability to discriminate faces by relying on configural information as shown by the dogs' performance in the blurred condition. While both naïve and expert dogs discriminated the owner above chance level, training, even if mainly planned to prime dogs' attention on face parts, allowed experienced dogs to perform near to perfection in the blurred condition. No significant differences were found between naïve and expert dogs in the grey-scale condition thus indicating that the effect of training experience was condition-specific.

## General Discussion

The present study provides the first evidence that pet dogs can discriminate isolated internal features of a human face. Actually, a number of recent studies focused on dog's discrimination or visual inspection of human faces without knowing if dogs' visual acuity certainly allows perception of feature details. Given our results, the specific pattern of face visual inspection and discriminability observed in those studies assumes higher or different value. For instance, the poor performance of dogs in discriminating human faces when only their inner parts (eyes, nose and mouth) are visible observed by Huber et al. [7] cannot be explained in terms of deficiencies of visual acuity.

Among internal features, the eyes region may be the most salient for human face discrimination by dogs. This result is in line with observations made in other species. In humans, presenting whole faces with the eyes concealed impairs the identification of kin [58] and gender [59] more severely than if other facial features are concealed. Similarly, individual conspecific recognition is impaired in chimpanzees when eyes are masked and in rhesus monkeys when both eyes and mouth are masked [60]. Finally, Kyes and Candland [61] found a visual preference for the conspecific's faces in which the eye region was visible in baboons. Overall these studies indicate that eyes are more important than other internal face features in face processing by primates. In dogs, the salience of internal face features was poorly investigated and, to the best of our knowledge, there is no study comparing salience or discriminability between internal features of the face. Using eye movement tracking, Somppi et al. [9] provided the only clear evidence that the human eyes region is a very salient feature for dogs. In agreement with our findings, this region is attracting nearly half of the relative fixation duration of the whole face area (hair and ears excluded). Whether the eyes region is at the first place in the hierarchy of face features saliency remain an open question. External features have been shown to contribute significantly to human face discrimination for humans [62]–[64], baboons [65], dogs [7] and sheep [66]. As previously cited, dogs seem to easily discriminate human face pictures when also the external cues (face profile, hair) are provided while performing very poorly when only the internal features of the face were visible [7]. While this seems to be in contrast with our results about eyes salience, methodological issues may account for this difference. First, our stimuli were adjusted in size to match that of the real heads and, not fitting entirely within the screen, external features were poorly represented in the pictures. Specifically, the hair outline was always absent, while face profile and ears were unevenly present. Second, the dogs of the present study were trained to discriminate internal features in three out of six training phases whereas in the other three phases internal or external feature could have been rewarded. Accordingly, our procedure draws the dogs' attention to the internal face features thus reducing salience of external features. Analogous effects of attention allocation on internal features salience are documented in humans while looking at other-race faces [67], [68].

Besides salience, lower physical properties (e.g. color, contrasts, shading) and spatial arrangement of facial features have an influence on face processing. The dogs of our naïve group were able to discriminate their owner face when configural information of internal features were readily available (i.e. grey-scale and blurred condition) while manipulation of even basic first-order spatial relationship (eyes are above the nose, which is above the mouth, etc.) by rotating faces 180° made them failing the discrimination. That means, even if dogs can discriminate single features, as proved by Experiment 1, this is not sufficient to allow discrimination of a face, when information about features configuration is varied. In humans, the relevance of configural information has been demonstrated by showing that even alterations of single parts are easier to detect when presented within the context of the face than when such parts are shown in isolation e.g., [15], [69]. Decrement of face discrimination abilities in inverted and scrambled conditions has been demonstrated, even if to a different extent, in many species, such as humans, e.g. [14], primates (reviewed in [70]), sheep [66] and pigeons [35]. In dogs, a deteriorative effect of the inversion on visual inspection of human face pictures has been previously found in a free viewing task [9] and using a visual paired comparison paradigm [11]. In this regard our results suggest that inversion affects the dog's ability to discriminate the owner's face, supporting what was indirectly suggested by comparison between familiar and novel face pictures [11]. Since all lower physical properties are unvaried in inversion manipulation, dogs seem to rely mainly on configural information when discriminating their owner's face. Moreover, configural information seems somehow to over-rule individual feature details as supported by the readiness of our naïve dogs to discriminate faces when features were blurred through pixilation.

The configural face bias observed in dogs may be grounded in an overall preference for global over local information in the processing of visual stimuli. In a previous study [71], although a large inter-individual variability was detected, dogs demonstrated a tendency to prefer the global over the local information when looking at hierarchical visual stimuli. The great interest in the eye area shown by dogs when looking at faces [9] is also, to some extent, indicative of configural face processing, since the eye region may actually provide more configural information than other features. That is because its central position may lead to an extraction of information about the whole face [72]. Notwithstanding, the present study does not permit to clarify if the crucial role of first-order spatial relationship of internal features is limited to face pictures. Racca et al. [11] failed to prove specific inversion effect for faces (of either dogs or humans) and there is no other evidence of face-specific configural processing by dogs. However, the authors discuss the lack of this finding to the very conservative methodology used for assessing the inversion effect and so dissimilarity of cognitive strategy in processing face by dogs warrants further investigation.

The role of specific experience on human face processing by pet dogs was the third aim of the present study. In contrast to the ‘naïve’ group, the ‘expert’ dogs were able to identify the owner in all test conditions, suggesting a facilitative effect of training on the dogs' ability to process human faces. It could be argued that training have exerted its facilitative effect by the simple exposition to the face pictures or their parts thus increasing the dogs' general familiarity with the stimulus and therefore their ability to discriminate the owner in different conditions. Nonetheless, the lack of an experience effect in the grey-scale condition suggests that training did not result in a general, unspecific improvement of their ability to discriminate the owner's picture, since the only unavailable information was the colour, a feature on which expert dogs had not been specifically trained on. The effect of training is well known in humans; training improves face discrimination and, in particular, reduces the other-race effect [50]. This effect is supposed to be a consequence of training on attention being directed to those features of other race faces that are useful for identification [73]. The distribution of eye movements is indeed critically affected by whether conspecific versus non-conspecific faces are shown [34]. This species specificity in face visual inspection has also been previously shown in dogs, who use a different gaze strategy while viewing human faces compared to dog faces [10]–[12]. It is then likely that training of our ‘expert’ dogs have modified their strategy for gazing at human faces, drawing their attention to more useful features as a consequence of previous training. Our results in the blurred condition imply that it is not only part-based processing that benefits from training, but that even the more readily used configural processing is easily enhanced by experience. Notably, even in humans the ability to use configural coding mechanisms appears to require expertise with a class of stimuli, e.g. [74], [75], and the relatively poor discrimination of faces from an unfamiliar race may well be the result of limited encoding of configural information for other-race faces [76], [77].

The present study has some limitations that warrant consideration. Firstly, participating owners were all females, and, accordingly, all the stimuli used in these experiments represented female faces. Although there is no direct evidence suggesting that dogs process female and male human faces differently, the fact that dogs could generalize the discrimination of human facial expressions only to novel faces of the same gender warrants caution [8]. Another possible limitation of the study is that, while our naïve dogs had not undergone the same specific experience that the experts had, they still had experience of living with humans. To which extent such exposure influenced the mechanisms observed in this study, cannot be determined from our data. Solid evidence for the importance of individual experience with the real 3D-referents of pictures for discrimination of the latter comes from pigeons [78]. It would be interesting to examine how dogs without or with very limited exposure to humans (e.g. feral dogs) would perform in the tasks presented in this study.

## Conclusions

In summary, visual processing of human faces by pet dogs seems to rely on configural information more than on information about face parts. Despite the great exposure of pet dogs to human faces in daily life, their ability to use primarily internal face features to discriminate even very related individual seems insufficient. Also the saliency of eyes may emerge to facilitate the extraction of global information about the whole face, rather than to gain detailed information about single parts. Far from being exhaustive, the present results encourage further studies on cognitive mechanisms underlying face processing in dogs. Among others, the salience of external face features, the face-specific role of the spatial relationships between features, the role of a different extent of exposure to human faces and the con-/hetero-specific effect need to be addressed to advance our understanding of the evolution of face processing abilities in mammals.

## Supporting Information

Dataset S1 (XLSX)
